# The Cancer Epitope Database and Analysis Resource (CEDAR): current capabilities and future directions

**DOI:** 10.3389/fonc.2026.1898826

**Published:** 2026-08-19

**Authors:** Zeynep Koşaloğlu-Yalçın, Ibel Carri, Daniel Marrama, Eve Richardson, Jason Greenbaum, Nina Blazseka, Randi Vita, Hannah Carter, Morten Nielsen, Bjoern Peters, Alessandro Sette

**Affiliations:** 1 Center for Vaccine Innovation, La Jolla Institute for Immunology, La Jolla, CA, United States; 2 Bioinformatics Core, La Jolla Institute for Immunology, La Jolla, San Diego, CA, United States; 3 Department of Medicine, University of California, San Diego, La Jolla, CA, United States; 4 Department of Health Technology, Technical University of Denmark, Lyngby, Denmark

**Keywords:** bioinformatics & computational biology, cancer epitopes, cancer immunology and immunotherapy, database, epitope prediction

## Abstract

Cancer epitopes, the molecular structures recognized by T and B cells at the tumor interface, are central to understanding antitumor immunity and developing immunotherapies. Yet despite the rapid growth of cancer immunology data, a comprehensive, continuously updated, and accessible resource for cancer epitope data has been lacking. The Cancer Epitope Database and Analysis Resource (CEDAR, cedar.iedb.org) was established in 2021 to fill this gap, providing curated experimental epitope data alongside a suite of cancer-specific computational tools for epitope prediction and analysis. Built on the validated infrastructure of the Immune Epitope Database (IEDB), CEDAR integrates cancer epitope data with biological, immunological, and clinical context, enabling researchers to explore immune recognition of tumors, identify candidate targets for immunotherapy, and benchmark prediction methods. Here we describe CEDAR’s current capabilities, report on progress in curation, database development, and tool availability, and outline the opportunities and challenges ahead for expanding its scope and utility to the cancer research community.

## 1 Introduction

Cancer epitopes are molecular structures recognized by the adaptive immune system through T-cell receptors (TCRs) and B-cell receptors (BCRs) or antibodies. When this recognition occurs, it initiates or modulates the immune responses capable of eliminating the cancer cells expressing these epitopes. Epitope-directed immune responses are mediated by diverse effectors, including CD4^+^ and CD8^+^ T cells and antibodies, engaging highly diverse TCR and BCR repertoires, which are linked to variable clinical outcomes (1).

Their central role in anti-tumor immunity has made cancer epitopes a major focus of both therapeutic developments and basic research. A range of immunotherapeutic modalities, such as immune checkpoint inhibitors, adoptive cell transfer, bispecific antibodies, and cancer vaccines, have been utilized with varying degrees of success (2, 3). In parallel, basic research continues to uncover novel epitope classes (4–6), characterize T- and B-cell responses at increasing resolution (7), and elucidate the mechanisms underlying immune recognition and evasion in cancer (8, 9). Cancer immunology research is generating an unprecedented volume of diverse immunological data, obtained from tumor sequencing, human leukocyte antigen (HLA) typing, epitope recognition assays, immune profiling of responding cells and clinical outcomes. It must be emphasized that experimental validation of epitope recognition does not automatically translate into a clinically viable immunotherapeutic target. Factors such as tumor-mediated immunosuppression, low surface presentation, or clonal heterogeneity can prevent therapeutic utility, highlighting the relevance of interconnecting diverse data types to understand and isolate truly actionable immune responses.

The molecular composition of cancer epitopes is remarkably heterogeneous, encompassing non-peptidic structures, including tumor-associated glycolipids, glycopeptides, and carbohydrate-based antigens arising from aberrant glycosylation (10), and a large breadth of peptidic epitopes derived from diverse protein sources. The latter include neoantigens (11) arising from genomic mutations, dysregulated RNA splicing, and post-translational modifications, as well as overexpressed self-proteins, cancer germline antigens, and oncoviral proteins. This heterogeneity, while challenging to study, offers unique opportunities to advance the understanding of cancer immunopathology and immunotherapy, highlighting the value of a centralized, regularly updated repository integrating the data available across all relevant studies.

The Cancer Epitope Database and Analysis Resource (CEDAR, cedar.iedb.org) was established in 2021 to address this gap directly. CEDAR leverages the infrastructure of the Immune Epitope Database (IEDB) (12–14), a long-established resource for epitope data in infectious diseases, autoimmunity, allergy, and transplantation. It expands its scope to include the cancer domain, which was previously outside IEDB’s focus. This extension allowed CEDAR to leverage validated curation processes, database architecture, and ontology integration while providing a dedicated, cancer-scoped interface tailored to the needs of the cancer research community. As of early 2026, CEDAR has curated human cancer epitope data from over 2,600 publications published since 2012, and its usage has grown year over year, reflecting increasing adoption by the cancer immunology community.

Here, we provide an updated description of CEDAR version 1.0, which transitioned to a fully functional release in early 2025. We summarize database content expansion and curation progress through early 2026, describe current query and analysis functionalities, compare CEDAR with related cancer epitope resources, and outline planned development priorities.

## 2 Resource construction

The CEDAR database was built on the existing infrastructure of the IEDB, which has been operational since 2003 and represents over two decades of development in epitope data curation, database architecture, and ontology design (12, 13). The IEDB’s core data model, curation pipeline, and community-supported ontology framework provided a validated foundation that CEDAR extended to the cancer domain. Fields and curation rules that were directly applicable to cancer epitope data were reused without modification, enabling structured cancer epitope curation from the outset. Cancer-specific fields were then identified and added, informed by structured input from an advisory board of cancer immunology experts. These new fields capture data elements specific to the cancer context, including antigen subtype classification (neoantigen, viral antigen, germline/self/host antigen), tumor sample features such as xenograft, carcinogen-induced, and genetically engineered models, cancer stage and histology using National Cancer Institute (NCI) Thesaurus terminology (15), mutation-level genomic annotations, and treatment and clinical outcome data. All terminology, both reused and newly defined, is grounded in community-supported ontologies from the Open Biological and Biomedical Ontology Foundry (16). The curation interface enforces field-level and inter-field validation rules to maintain data integrity at scale. The CEDAR query and reporting interface was developed iteratively based on user feedback, building on the IEDB’s query framework while adding cancer-specific search parameters not present in the IEDB, including antigen subtype, mutation, cancer type and stage, and receptor sequence search. A detailed account of the IEDB curation processes on which CEDAR builds is available in prior publications (14, 17–19).

In parallel, the CEDAR Analysis Resource was built on the Next-Generation IEDB tools platform (20), which provides the shared infrastructure for modular tool integration and reproducible pipeline construction. Cancer-specific tools were developed within this framework based on direct input from cancer immunology researchers, with inputs and outputs iteratively refined to reflect the specific data types and workflows encountered in cancer research, such as variant call files, paired mutant and wild-type peptide sets, and patient HLA genotypes. All cancer-specific tools are tagged within the platform, allowing users to filter and identify them easily alongside the broader suite of general immunological prediction tools (21).

## 3 Results

### 3.1 CEDAR development milestones and progress since inception

**Figure 1:**
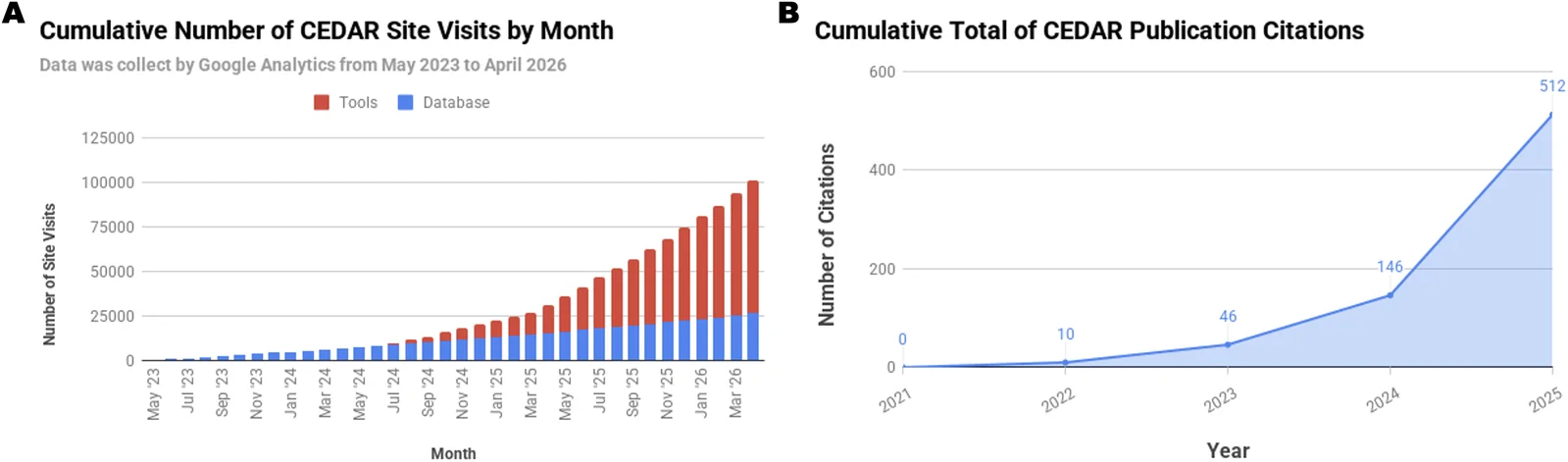
**(A)** Cumulative monthly site visits to the CEDAR database and tools. Site visit data were collected using Google Analytics 4 and reflect the number of sessions per month from May 2023 onward. A session is defined as a period of continuous user interaction with the website. Data from the CEDAR database and tools sites are tracked separately and shown as distinct series. **(B)** Cumulative citations to CEDAR publications.

CEDAR was first described in August 2021 (22), with a web-accessible alpha version available in early 2022 (23). A stable beta version was released in 2024, and a fully functional 1.0 version was announced in early 2025. Since its launch, the resource has seen consistent growth in both usage and community recognition, averaging over 735 monthly database visits per month and accumulating over 500 citations to CEDAR publications by December 2025 (**Figure 1**; **Supplementary Figure 1**). Traffic to the shared Next-Generation IEDB tools platform, which hosts CEDAR’s cancer-specific tools alongside general immunological prediction tools, averaged 3,392 monthly visits over the same period. These metrics suggest growing recognition of CEDAR as a resource for cancer epitope data and tools within the immuno-oncology community.

**Figure 2:**
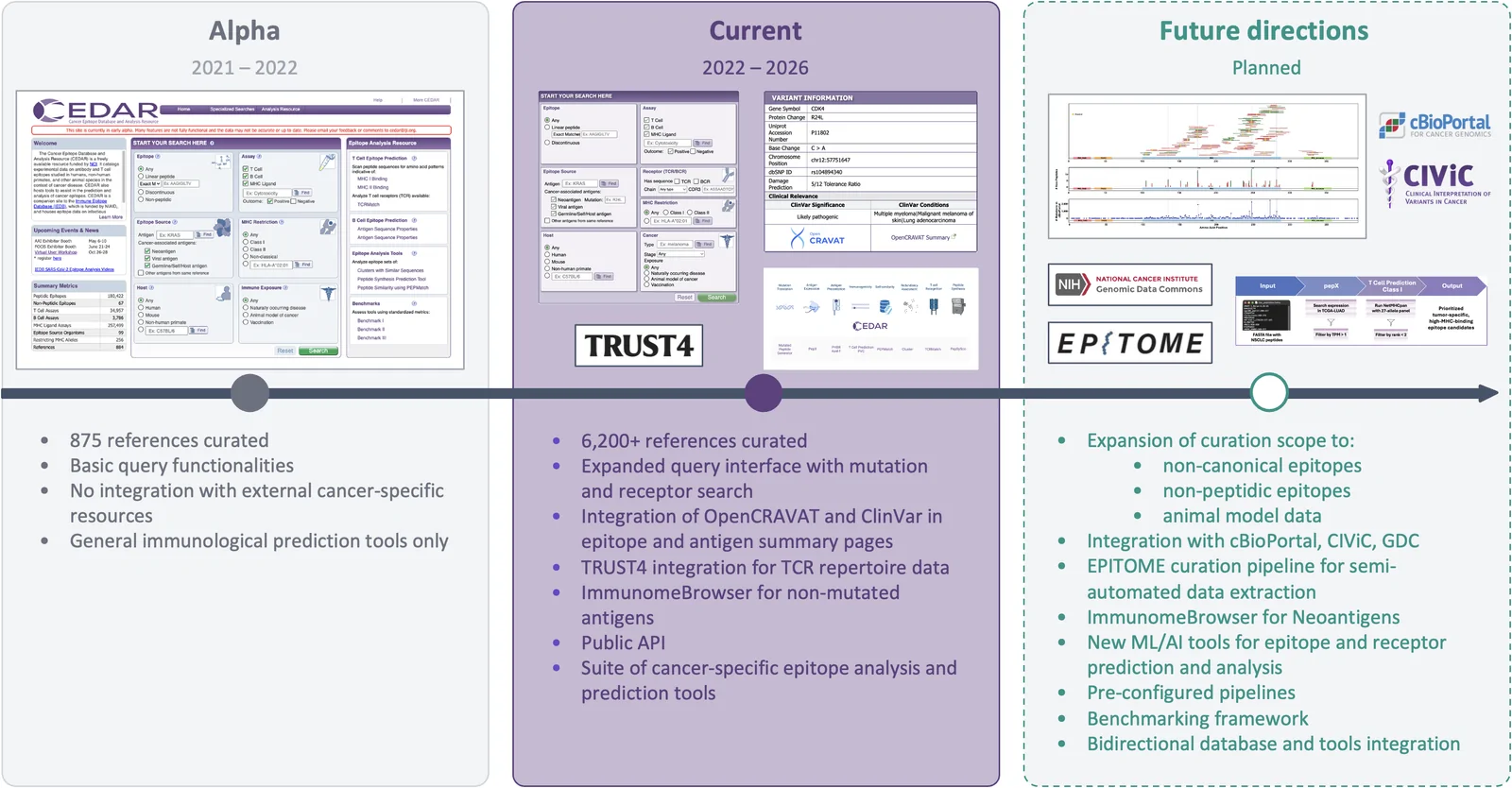
CEDAR development timeline. Overview of key milestones and planned developments across three phases: the alpha release (2021 to 2022), the current version (2022 to 2026), and planned future directions. Representative interface screenshots and resource logos are shown for each phase.

Since the alpha release, the database has grown from 875 curated references to 6,203, with over 440,000 epitope structures, 1.5 million assays, and 60,000 receptors as of July 2026 (**Table 1**). The interface has matured substantially, evolving from basic query functionality into a fully featured platform with cancer-specific search fields, mutation and receptor search panels on the homepage, antigen and neoepitope summary pages, customizable data exports, and a public API enabling programmatic access to curated data (**Figure 2**). Variant-level annotations from OpenCravat (24) and ClinVar (25) are now integrated directly into neoepitope summary pages, connecting curated epitope records to genomic and clinical context.

**Table 1:** Summary metrics for the CEDAR database as of July 2026.

Metric	Counts
Epitopes	445,931
Publications	6,203
Cancer Types	26
Neoantigen Assays	45,132
Viral Antigen Assays	98,808
Germline Antigen Assays	1,385,215
MHC Class I Assays	1,035,230
MHC Class II Assays	384,729
T-cell Assays	112,186
B-cell Assays	82,941
Positive Assays	1,413,496
Negative Assays	115,659
Human Assays	1,478,648
Animal Model Assays	1,386
Receptors	60,104

At launch, CEDAR provided access only to general immunological prediction tools inherited from the IEDB platform, such as MHC binding prediction, which were not designed with the cancer context in mind. Since then, a dedicated suite of nine cancer-specific tools has been made publicly available (21). These tools can be used independently or assembled into custom analysis pipelines. An integration with TRUST4 (26) has been established, enabling researchers to link immune receptor sequences reconstructed from tumor RNA-seq data directly to epitope specificities curated in CEDAR and the IEDB.

To further support scalable curation, an AI-assisted curation pipeline (EPITOME) has been developed, with a proof-of-concept demonstrating its potential to accelerate structured data extraction from the literature (27), and full deployment within the CEDAR curation workflow is planned (**Figure 2**). Looking ahead, the development priorities outlined in this manuscript include broader curation coverage of non-peptidic epitopes, animal models, and non-canonical neoepitope classes; new tools and pre-configured pipelines; a transparent benchmarking framework; and deeper interoperability with cBioPortal (28), Clinical Interpretation of Variants in Cancer (CIViC) (29), and the Genomic Data Commons (30).

Several existing repositories capture portions of the cancer epitope landscape (**Supplementary Table 1**). The TANTIGEN 2.0 database (31) provides curated epitope and ligand elution data for a range of cancer antigens, but has not been updated since 2021, and lacks integration with clinical data. NEPdb (32) provides curated neoantigens with associated receptor data but excludes non-mutated antigen classes and does not support receptor-focused searches, with coverage limited to 2019. CEDAR addresses these limitations by providing continuously updated, comprehensive coverage across antigen classes, effector types, and clinical contexts, built on a curation methodology validated over two decades of IEDB development and refined through direct engagement with the cancer research community.

### 3.2 CEDAR database ontology, query, and reporting functionalities

The CEDAR database is organized around key data elements that facilitate data organization and enable precise querying. These include: (i) epitope structure, encompassing amino acid sequence and post-translational modifications such as phosphorylation and glycosylation; (ii) epitope origin, defined by source protein/antigen; (iii) characteristics of the host associated with epitope responses, including species, age, sex, strain or ethnicity, and associated MHC type; (iv) features of the tumor sample, or model, such as xenograft, carcinogen-induced, and genetically engineered or spontaneous tumors; (v) characteristics of B- and T-cell immune response effectors, including antibody class and subclass, effector cell source, such as ex-vivo, short-term cultures, stable cell lines, and, if available, TCR and antibody sequences; (vi) assay details, including modality and functional outcome of effector responses, including epitopes and cancer cell recognition; and (vii) information source.

**Figure 3:**
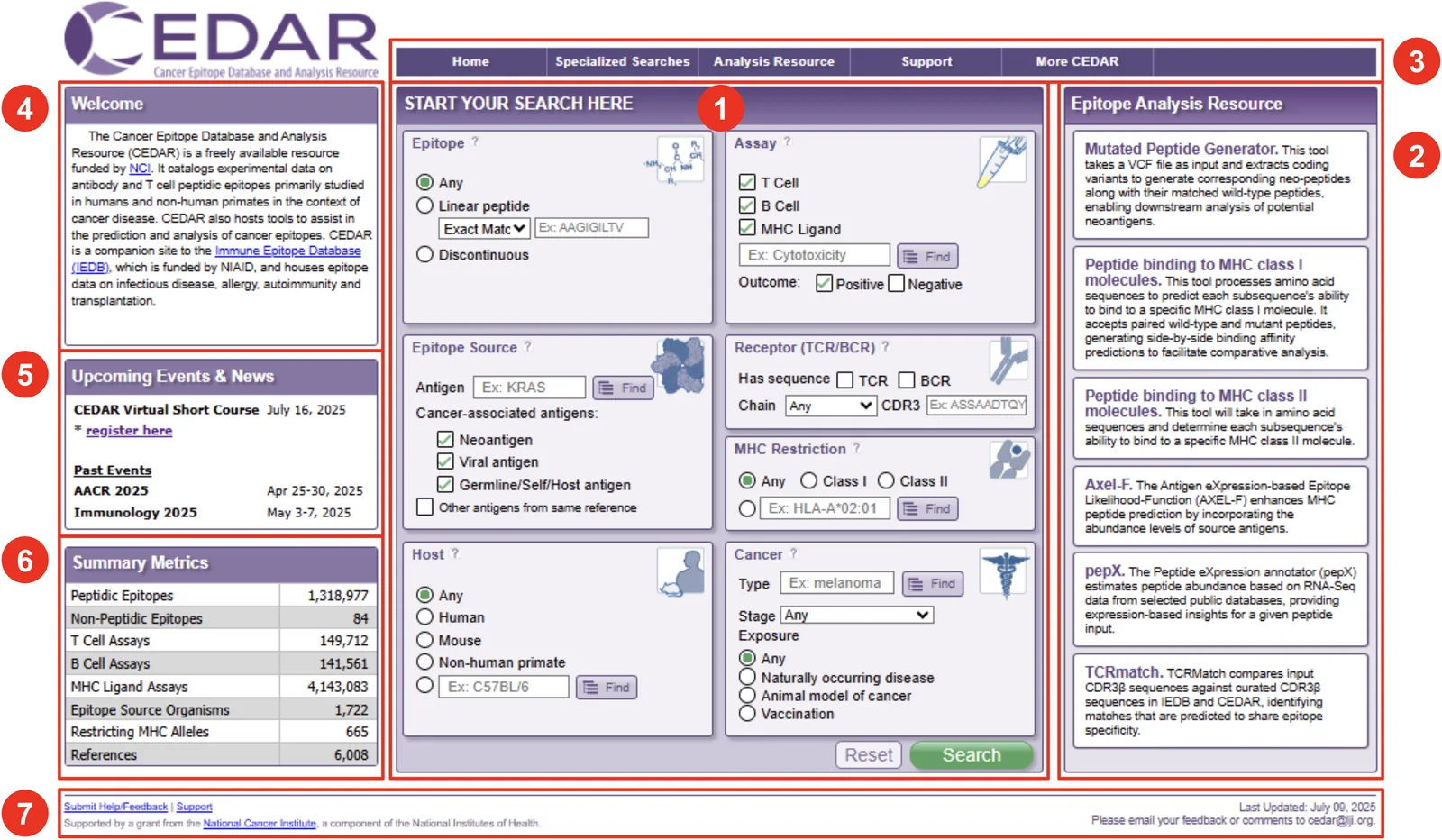
CEDAR homepage accessed at cedar dot iedb dot org. (1) start your search here panels, (2) analysis resource, (3) menu, (4) welcome, (5) upcoming events & news, (6) summary metrics, and (7) footer links.

All terminology is grounded in community-supported ontologies or broader external databases, ensuring precise, consistent, and interoperable data representation. For example, the NCBI taxonomy (33) database is used to capture the precise strain from which viral epitopes originate, including synonymous names, enabling queries as specific as a particular isolate of HPV16 or as broad as all epitopes derived from the genus Alpha papillomavirus. Cancer-specific fields draw on standards such as the NCI Thesaurus (15) for cancer stage classification. The CEDAR web query interface provides access to all of these data elements (**Figure 3**).

**Figure 4:**
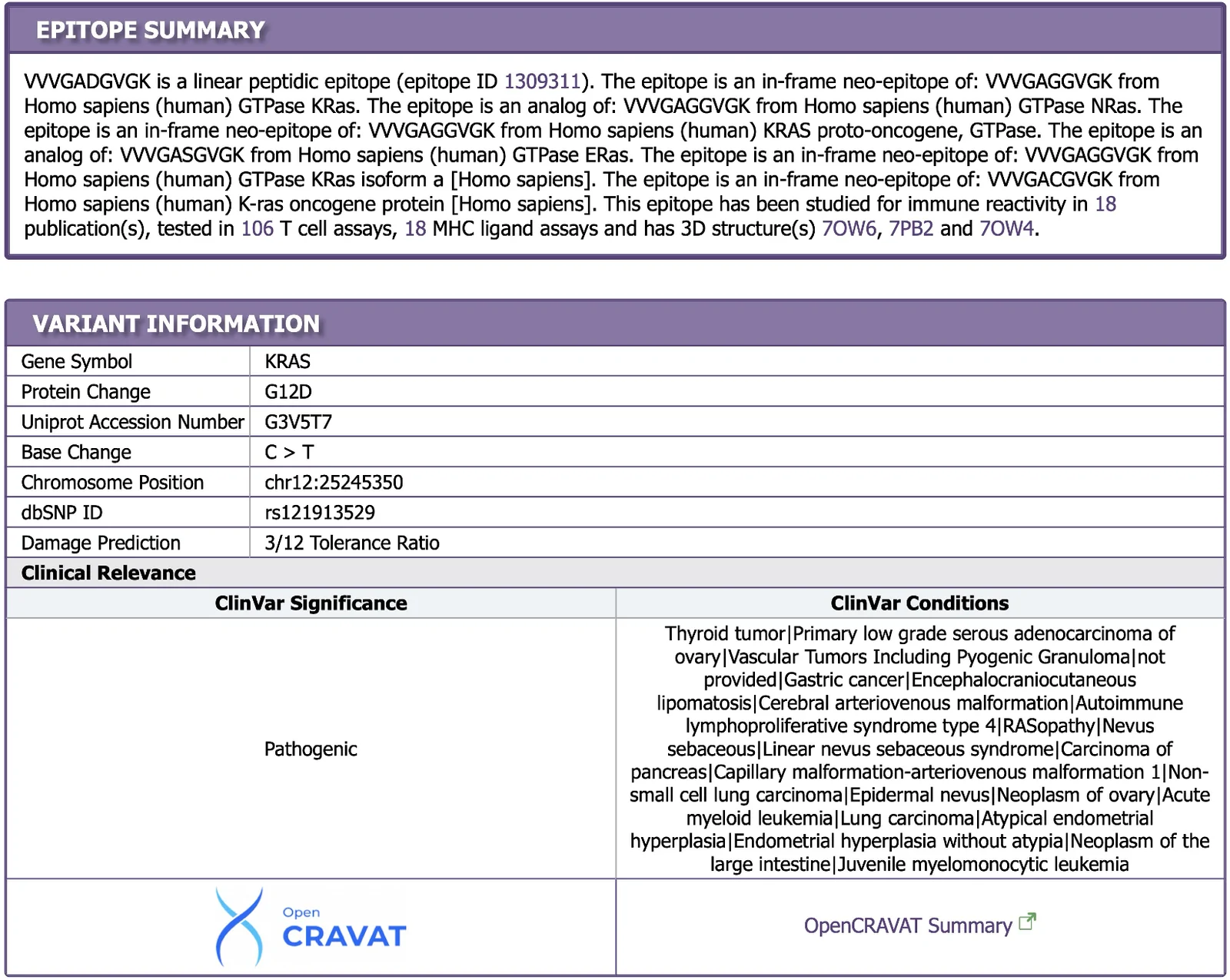
Neoepitope summary page developed in collaboration with OpenCRAVAT and ClinVar.

Recent efforts have focused on expanding cancer-specific annotations through integration with external resources. The Epitope Summary pages for neoantigens now include variant-level information from OpenCravat (24), such as base changes and chromosomal coordinates, predicted functional effects, and ClinVar (25) annotations (**Figure 4**).

All query results can be downloaded directly from the results display across all tabs, including epitopes, antigens, assays, receptors, and references, in tabular format with custom column selection. Complete database exports are also available in various formats through the bulk download page, accessible from the CEDAR homepage. To further support large-scale and automated data access, we recently launched the CEDAR Application Programming Interface (API), built on a PostgREST platform that provides transparent programmatic access to the backend database tables. Comprehensive public documentation is available through a dedicated Discourse help article (https://discuss.iedb.org/t/application-programming-interface-api/220) and an interactive Swagger interface (https://cedar-api.iedb.org/docs/swagger/), including practical examples for integrating CEDAR data into automated workflows using Python. Together, these options ensure that CEDAR data are accessible to users across the full spectrum of technical expertise, from manual browsing and download to large-scale programmatic integration.

#### 3.2.1 Future enhancements for query and reporting functionalities

The following features are planned for future development and are not yet available in the current release. Integration with CIViC (29) and cBioPortal (28) will enable users to seamlessly access complementary genomic and clinical annotations directly from CEDAR epitope pages, including variant functional impact, recurrence across tumor types, therapeutic associations, and links to patient outcome data.

Additional annotation layers are planned to capture other antigen-associated properties, such as subcellular location and biological function based on GeneOntology (34, 35), expression in pre-malignant tissues, frequency and magnitude of expression across tumor types (28, 36), and cancer cell lines (37), and the expression profiles of epitope source antigens in diverse healthy tissues across developmental stages (38). For epitopes derived from non-self sources, such as oncoviral proteins, the specific antigen of origin will also be recorded, along with information on endogenous retroelement antigens, such as long terminal repeats or endogenous retroviruses (39).

Query functionalities will be further expanded to accommodate the full diversity of neoantigen-generating mechanisms. Current annotations cover single-nucleotide variants (SNVs) and in-frame insertions and deletions (indels). Future extensions will include more complex neoantigen sources such as chimeric RNAs and noncanonical transcripts, as well as processes like alternative RNA splicing, transposable element activation, and post-translational modification. Each could be categorized by its predicted functional impact, leveraging the Sequence Ontology (40) and annotated for biological consequences, including effects on protein function, structure, localization, predicted surface accessibility, and dysregulated expression (41–44).

**Figure 5:**
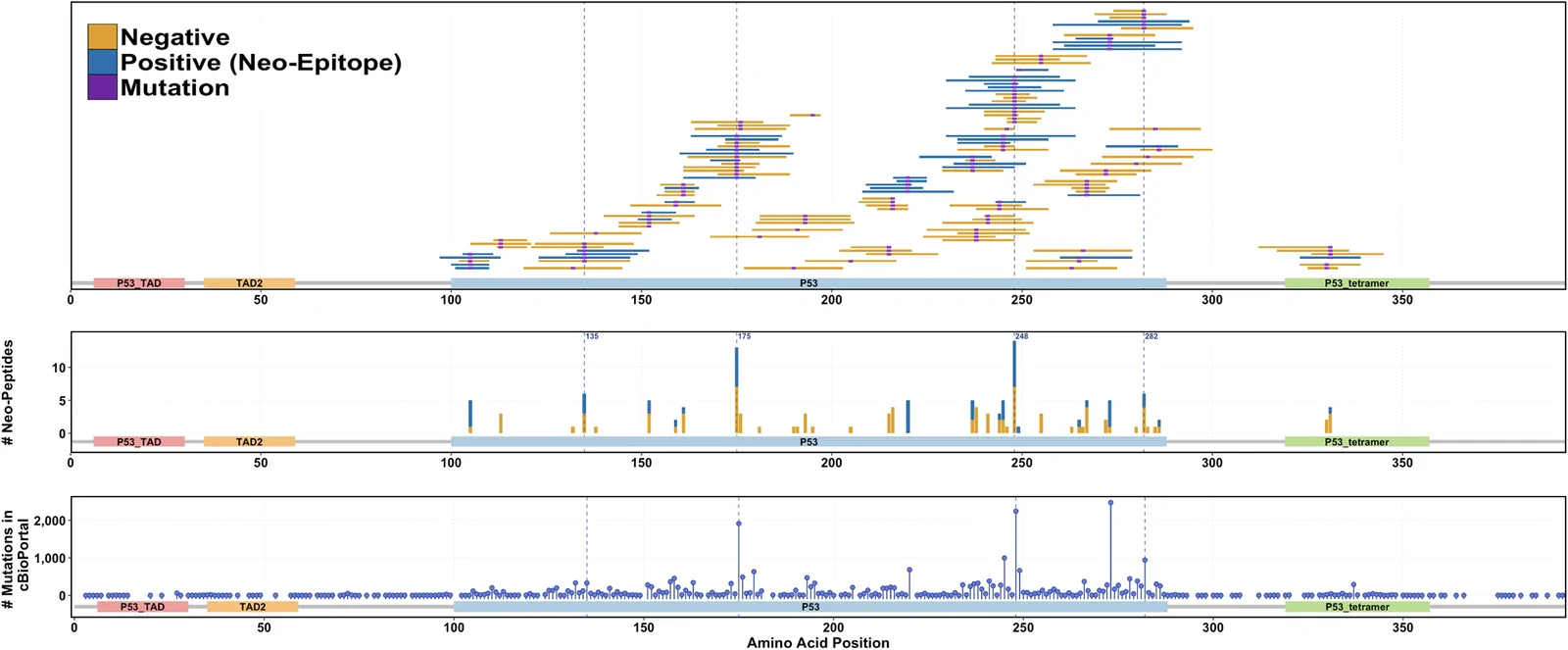
Prototype for neoantigen immunomebrowser, including mutational frequencies from cBioPortal. Reproduced by Sette et al. (2025) (45) licensed under CC BY 4.0.

A Neoantigen ImmunomeBrowser, currently in prototype, will extend the existing ImmunomeBrowser functionality to display point mutations within antigen sequences alongside associated immunogenicity data, and will incorporate mutational frequency data from cBioPortal to connect epitope-level immunogenicity to population-scale genomic context (**Figure 5**).

### 3.3 Curation of immune epitope data from literature relevant to cancer immunology

**Figure 6:**
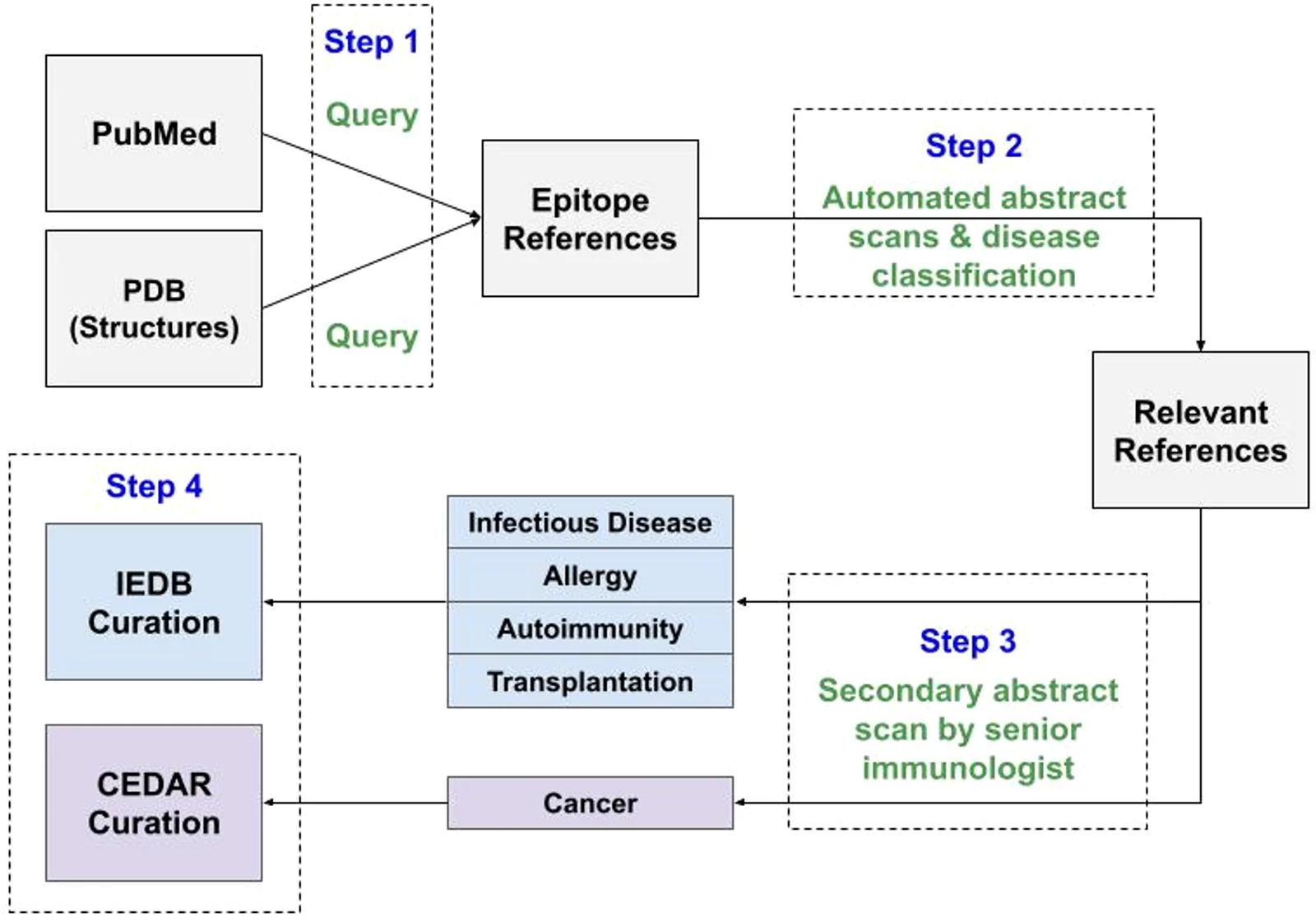
Workflow for identifying curatable journal articles.

The CEDAR curation pipeline builds on a literature monitoring strategy developed and continuously optimized over two decades in the context of the IEDB (14, 17, 19, 46–50), and extended here to the cancer epitope domain (22, 23). In this workflow (**Figure 6**), PubMed and the Protein Data Bank are monitored biweekly using broad, intentionally comprehensive keyword queries designed to retrieve all references containing immune epitope information, a strategy that has identified over 278,000 references from approximately 38 million PubMed articles (Step 1). Abstracts are then evaluated in two stages: automated text classifiers trained on previously curated records assign each abstract to one of several broad disease categories including cancer, infectious disease, autoimmunity, allergy, and transplantation (17, 51, 52), and a senior immunologist performs a secondary scan to confirm category assignments. References are considered within scope if they provide novel experimental data, describe epitope molecular structures with sufficient precision, and fall within the cancer category; review articles and studies in which epitopes serve only as markers or tags are excluded (Step 2). Full-text articles that pass this filter are assigned to doctoral-level curators for structured data extraction, following detailed curation guidelines (17, 48), and all curated records undergo peer review before becoming publicly visible (Step 4). Within the cancer category, references are further subdivided into subcategories grouping similar antigen types and cancer contexts, and prioritized in reverse chronological order to ensure the most recent literature is captured first. The pipeline has been continuously refined to accommodate evolving data types, including receptor repertoire sequencing data and neoepitope features such as frameshifts and splice variants.

Data quality is maintained through multiple complementary mechanisms. All curated records undergo peer review by a second doctoral-level curator before becoming publicly visible, providing a systematic check on data accuracy prior to each database release. During data entry, the curation system enforces both field-level validation rules, ensuring correct data type and format, and inter-field validation rules that check content consistency across related fields. For example, the system flags incompatible combinations such as an MHC-tetramer staining procedure specifying antigen-presenting cells, or a breast cancer diagnosis assigned to a non-mammalian host. Data refinement is an ongoing process guided by user feedback, internal team review, and validation rule outputs, with identified errors corrected systematically and change requests consolidated through biannual formal analyses. The curation manual, a publicly accessible living document (17), is updated continuously to reflect evolving standards and cancer-specific curation scenarios as they arise.

Full methodological details of the curation pipeline, including the document classification approach, inter-field validation rules, and quality control procedures, are described in prior publications (14, 19, 46–50, 53).

#### 3.3.1 Progress to date in the curation of cancer epitopes

Cancer-related publications account for 14.4% of all curatable epitope references identified through the literature monitoring pipeline. Within this subset, records are organized into subcategories grouping similar antigen types and cancer contexts and prioritized in reverse chronological order to ensure the most recent literature is captured first. As of early 2026, CEDAR provides complete coverage of cancer epitope-specific human immune response data published since 2012, spanning over 2,600 curated publications, with an annual throughput of approximately 650 references. This includes dedicated coverage of prostate cancer antigens and neoepitopes arising from SNVs and in-frame indels, which served as initial prototype categories representing non-mutated and mutated antigen classes, respectively (45, 54).

Each curated record consists of a structured set of data fields describing the epitope’s molecular structure, its source protein and organism, and the host in which the immune response was studied. If provided in the original publication, disease type and stage, effector cell populations, assay methodology, and response magnitude are also recorded, along with the sequences of any epitope-specific TCRs or BCRs identified in the study. All terminology is standardized using community-supported ontologies, ensuring consistency across records and interoperability with external resources (53).

#### 3.3.2 Application of machine learning techniques to partially automate general curation

We have engaged the Artificial Intelligence (AI) and Natural Language Processing (NLP) communities since 2006 to further automate our curation processes. This experience has taught us that, while specific tools may perform well in general information retrieval, adapting them to our curation processes poses significant challenges. A persistent problem has been that traditional NLP tools disregard the figures and tables that contain a substantial amount of the information to be captured for CEDAR curation. Furthermore, our use case for comprehensive curation of individual documents with an exceptionally low tolerance for error is not well-suited for traditional NLP approaches.

Recent AI advances have revolutionized the ability to process free text data using Large Language Models (LLMs) trained on massive amounts of text. We have been working to utilize these advances in the CEDAR curation process. We implemented a multimodal document ingestion and Question-Answering (QnA) pipeline that ties traditional Optical Character Recognition (OCR) and text matching with Vision-Language Model (VLM) capabilities. The system, which we call EPITOME, implements three-stage processing: visual element extraction from PDFs, regex-based epitope and MHC molecule identification, and contextual indexing that links peptide sequences, MHC molecules, and assays to their locations across text, tables, and figures. This indexing is used to supply context for further VLM QnA.

As a preliminary proof-of-concept we applied EPITOME to 251 previously curated references reporting 6,418 Major Histocompatibility Complex (MHC) Class I binding assays. These initial results demonstrate that open-source VLMs in a zero-shot pipeline are capable of recognizing 100% of the assay responses in nearly three-quarters of the publications, highlighting their potential to accelerate scientific biocuration. However, a curator-in-the-loop oversight remains crucial to enhance overall system accuracy. EPITOME is not yet integrated into the production curation workflow. Full deployment of EPITOME within the CEDAR curation workflow is planned as a near-term priority. The full results of this foundational work are currently under review (27).

#### 3.3.3 Future directions for the curation of cancer epitopes

While CEDAR curation currently focuses on peptide-based epitopes, non-peptidic epitopes represent an important yet underrepresented component of the cancer-associated epitope repertoire. These include altered glycolipids, glycopeptides, and carbohydrate-based antigens arising from aberrant glycosylation, dysregulation of lipid metabolism, or oncogenic viral transformation (55–57). Expanding CEDAR curation to capture these epitope classes and making them searchable through the query interface represents an important future direction, as non-peptidic antigens are central to humoral antitumor responses and underpin several diagnostic and therapeutic antibody strategies (58). CEDAR has thus far achieved comprehensive coverage of neoepitopes arising from short somatic mutations within protein-coding sequences, including SNVs and in-frame indels, which are among the most extensively validated due to their reliable detection via next-generation sequencing and the availability of established computational pipelines (11). An important future direction is expanding this coverage to neoepitopes arising from additional mechanisms that generate immunogenic neoantigens, including frameshift mutations, gene fusions, splice variants, premature start-gain and stop-loss mutations that activate untranslated regions, transposable element activation, noncanonical transcripts, chimeric RNAs, and post-translational splicing and modifications (4, 5, 59–63). Animal model data and pre-2012 literature also represent additional opportunities to substantially broaden the historical and experimental breadth of CEDAR.

### 3.4 Cancer epitope prediction and analysis tools

**Figure 7:**
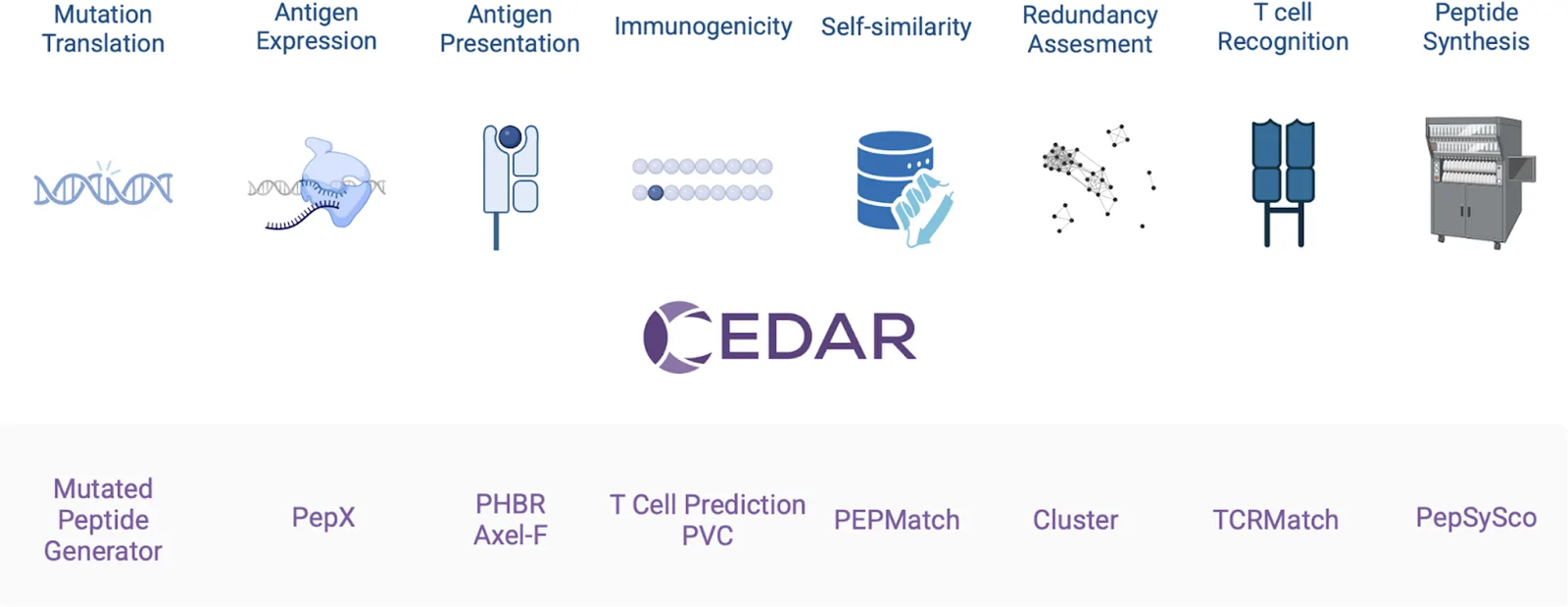
Overview of tools available in CEDAR that support critical steps in cancer epitope identification. Reproduced by Cari et al. (2026) (21) licensed under CC BY 4.0.

CEDAR offers a dedicated suite of tools for cancer epitope prediction and analysis that address the specific needs of cancer immunology research (**Figure 7**). All nine tools described below are currently available at the URLs listed in **Supplementary Table 2**. These tools are integrated with the broader IEDB analysis resource (20), but offer distinct cancer-focused functionalities. A key strength of this integration is that CEDAR can incorporate general epitope prediction capabilities, such as MHC-binding predictions, directly into cancer-specific applications and modular pipelines.

CEDAR tools currently support critical steps in cancer epitope identification (**Figure 7**; **Supplementary Table 2**). The Mutated Peptide Generator (MPG) automates the extraction of mutant and corresponding wild-type peptide sequences from genomic variant calls. The Peptide Variant Comparison Tool (PVC) enables side-by-side evaluation of MHC binding, elution, and immunogenicity predictions for mutant and wild-type peptide pairs. Axel-F (64) estimates the likelihood of epitope presentation by integrating source antigen abundance levels, validated by demonstrating a significant correlation between antigen abundance and T cell recognition in independent datasets. PepX (65) annotates peptides by identifying their potential source proteins and estimating peptide abundance from aggregated public transcriptomic datasets, validated against tissue-specific expression profiles. The Patient Harmonic-mean Best Rank tool (PHBR) (66) ranks peptides based on predicted MHC binding across a patient’s full HLA genotype, validated in the context of oncogenic mutational landscapes. PEPMatch (67) enables rapid identification of sequence-similar peptides across reference proteomes and was benchmarked for speed and accuracy across proteome-scale datasets. TCRMatch (68) predicts T cell receptor specificity based on sequence similarity to previously characterized receptors and was validated against curated TCR:epitope pairs from IEDB and CEDAR. A sequence clustering tool (69) reduces redundancy in peptide datasets and was validated for immunological applications. PepSySco (70) predicts the success of Fmoc-based peptide synthesis and was validated against experimental synthesis outcomes. These tools can be used independently or assembled into custom pipelines that mirror the biological steps of antigen processing and immune recognition (21).

#### 3.4.1 Future developments and goals for CEDAR prediction and analysis tools

The following capabilities are planned and not yet available in the current release. Building on the current CEDAR tools infrastructure, planned efforts will focus on maintaining and enhancing existing cancer-specific epitope prediction tools, expanding functionalities based on user requests, and establishing benchmarks to guide tool design and use. Existing tools will be continuously maintained through bug tracking, automated testing, and monitoring systems, ensuring reliability and rapid response to issues. Several new tools and capabilities are planned to address remaining gaps in cancer epitope analysis. Novel cancer-specific prediction tools and integrated analysis pipelines will be developed, incorporating state-of-the-art machine learning and AI methods to evaluate epitope immunogenicity, clinical relevance, and biomarker potential. Pre-configured pipelines tailored to common cancer immunology research scenarios will combine existing CEDAR tools into ready-to-use workflows for tasks such as neoepitope discovery from variant call files, combined HLA class I and class II binding and immunogenicity assessment, and expression-based antigen prioritization. These pipelines will include default parameters, example datasets, and interactive visualization options, lowering the barrier for less-experienced users while preserving the flexibility for advanced users to modify parameters or incorporate additional tools into custom workflows.

A transparent benchmarking framework for cancer epitope prediction tools is also planned. Numerous computational tools for cancer epitope prediction have been published and continue to emerge (71–73), with newly published tools typically reporting internal benchmarking results that cannot be independently verified. CEDAR will address this by providing curated benchmark datasets, such as sets of MHC class I-presented neoepitopes with experimentally confirmed recognition status, alongside automated live benchmark pipelines that generate predictions on these datasets immediately prior to their public release. This approach extends benchmarking infrastructure previously established for MHC class I (74) and class II (75) binding predictions in IEDB to the cancer epitope context and will provide regularly updated tool recommendations to guide both developers and end users.

Finally, tighter integration between the CEDAR database and analysis tools is planned. Currently, data from CEDAR cannot be directly accessed through the tools website, and query results from the database cannot be seamlessly passed to the tools for downstream analysis. Addressing this limitation will enable peptide sets returned by database queries to be sent directly to the tools site as input for prediction and analysis. Conversely, users who have performed predictions on the tools side will be able to check the experimental validation status of their candidate peptides against curated CEDAR records, with direct links to the corresponding experimental evidence. This bidirectional connectivity will enable seamless end-to-end workflows that move fluidly between curated experimental data and computational analysis.

### 3.5 CEDAR’s engagement with the cancer research community

CEDAR has been actively promoted to the broader cancer immunology community through multiple channels. To date, 28 peer-reviewed articles have been published, over 50 talks and posters have been presented at scientific meetings, and 12 promotional booths have been hosted at conferences, including the American Association for Cancer Research (AACR), the American Association of Immunology (AAI), and the Festival of Biologics (FoB). An annual CEDAR User Workshop, held online, attracted 410 registrants.

Community uptake is tracked through Google Analytics (76) and internal server logs, monitoring session volume, user origins, and visit duration, alongside citation analyses to assess the reach of CEDAR publications. Google Analytics 4 anonymizes all user data by default and automatically excludes bot traffic; no personally identifiable information is collected or retained. CEDAR operates under a privacy policy, which governs all data collection practices and is publicly accessible on the website. The geographic distribution of active users across the reporting period is provided in **Supplementary Figure 2**), reflecting broad international adoption of the resource.

CEDAR has also established a growing network of integrations with complementary cancer informatics resources. Linkage to OpenCRAVAT (24) enables high-level variant annotation and interpretation of neoantigen-related mutations curated in CEDAR. In collaboration with pVACtools (77), a systematic comparison of widely used approaches for cancer neopeptide generation identified sources of inconsistency and established best practices (78). Integration with TRUST4 (26) connects TCR and BCR repertoire data to validated epitope specificities in CEDAR and IEDB. Additionally, PROTEAN-CR (79) uses CEDAR to validate peptide presentation across proteomic and antigen-processing datasets, and iPepGen (80) leverages CEDAR’s curated data for peptide design and optimization.

To support this growing user base and establish a transparent mechanism for error reporting and data corrections, we recently launched a community platform (https://discuss.iedb.org/). Powered by Discourse, this portal hosts a dedicated, interactive CEDAR Knowledgebase and serves as the primary repository for user help articles and documentation. The platform transitions support from private communications to a public forum where users can ask questions, report data anomalies, and submit feedback. Because all threads, curation resolutions, and bug fixes are open to the public, the broader community can actively read, track, and contribute to ongoing technical discussions, ensuring an iterative and community-driven approach to data quality control.

To illustrate how CEDAR supports cancer immunology research in practice, a detailed step-by-step protocol with worked examples has been published separately (81). As one example, a researcher investigating recurrent mutations in cancer can enter a specific neoepitope sequence directly into the query interface and retrieve all curated experimental evidence for its immunogenicity, including MHC binding assays, T cell recognition data, and links to the original publications, enabling rapid assessment of a candidate before committing to experimental validation. As another example, a researcher exploring viral antigen targets for head and neck cancer immunotherapy can filter by cancer type, antigen subtype, and assay outcome in a single query, returning a downloadable list of HPV-derived epitopes with confirmed T cell recognition that can directly inform target prioritization for vaccine or adoptive cell therapy design.

## 4 Discussion

Cancer epitope data are being generated at an unprecedented scale, spanning tumor sequencing, HLA typing, epitope recognition assays, and detailed immune profiling across diverse patient populations. Yet the informatics infrastructure for organizing, accessing, and analyzing these data in a cancer-specific context has lagged behind. CEDAR was designed to address this gap, and the work described here reflects both the progress made and the substantial opportunities that remain.

Beyond the database, the CEDAR Analysis Resource provides a suite of publicly available, transparent, and broadly applicable computational tools. A major use case is the construction of neoepitope prioritization pipelines that move from somatic mutation calls through MHC binding prediction, antigen processing assessment, and T cell reactivity evaluation. General-purpose epitope prediction tools from the IEDB underpin these workflows, which are now customized for the cancer context and assembled into modular, reproducible pipelines. This addresses a persistent problem in the field, where published pipelines are often proprietary, difficult to access, or buried in **Supplementary Materials**. Nearly half of all traffic to CEDAR and IEDB is directed to the tools site (**Figure 1**), consistent with strong community interest in cancer-specific computational analysis capabilities. Importantly, the CEDAR Analysis Resource supports and fuels active epitope discovery, encouraging the experimental testing of novel candidates that ultimately populate and expand the CEDAR database. In turn, the database provides the essential training and validation material required to refine these computational tools. This creates an opportunity for a positive feedback loop between data and tools that may contribute to a deeper understanding of cancer epitope immunogenicity and inform the development of improved immunotherapies.

CEDAR exemplifies the power of interoperability between cancer informatics resources. Almost all data are stored using ontologies from the Open Biological and Biomedical Ontology Foundry (16), including the Ontology for Biomedical Investigations (82), the Cell Ontology (83), and Uberon (84). These ontologies ensure a precise definition of terms, enable search by commonly used synonyms, and provide a clear delineation of a hierarchical term structure and semantic integration with other resources, thereby increasing the Findable, Accessible, Interoperable, and Reusable (FAIR) nature of CEDAR. Furthermore, CEDAR database queries and tools were designed to be easy to integrate into other cancer-specific resources through an API.

Interoperability with the broader cancer informatics ecosystem is a key area of planned expansion. CEDAR’s ontological foundation already enables semantic integration with external resources, and a public API now allows programmatic access to curated data. Planned integrations with cBioPortal, CIViC, and GDC will further connect epitope-level data to genomic variants, HLA genotypes, gene expression profiles, and clinical metadata, enabling CEDAR to serve as a hub for comprehensive cancer immunity analyses. These integrations reflect a broader trend in cancer informatics toward federated, interoperable ecosystems in which resources complement rather than duplicate one another, and in which a researcher can move fluidly between epitope data, genomic context, and clinical outcomes within a single analytical workflow.

Taken together, CEDAR represents a comprehensive and actively maintained resource for cancer epitope data, and the expansions described here in curation scope, query functionality, analytical tools, and external interoperability reflect the directions in which the field itself is moving. As the volume and diversity of cancer epitope data continue to grow, so too does the value of a resource that makes them easily accessible to the broader scientific community.

## Supplementary material

Supplementary Figure 1 CEDAR monthly website traffic by quarter, showing site visits to the CEDAR database and the shared Next-Generation IEDB tools platform from May 2023 through Q1 2026. Data were collected using Google Analytics 4 and reflect the number of sessions per month, aggregated by quarter. Bot traffic is excluded automatically via GA4.

Supplementary Figure 2 Geographic distribution of active CEDAR database users by country, based on Google Analytics 4 data collected from May 1, 2025 to April 30, 2026. The top seven countries by active user count are shown alongside a global heat map of user distribution. Data reflect visits to the CEDAR database site and are anonymized by default; no personally identifiable information is retained.

Supplementary Table 1 Comparison of CEDAR with related cancer epitope and immunoinformatics resources.

Supplementary Table 2 Cancer epitope analysis and prediction tools available in CEDAR. For each tool, the required input, expected output, and URL are provided. Tools are accessible through the Next-Generation IEDB tools platform (nextgen-tools.iedb.org) or the IEDB tools site (tools.iedb.org). All tools are freely accessible without registration.
